# Self-harm, Suicide, and ICD-11 Complex Posttraumatic Stress Disorder in Treatment-Seeking Adolescents with Major Depression

**DOI:** 10.1007/s40653-024-00655-0

**Published:** 2024-08-21

**Authors:** Huanzhong Liu, Grace W.K. Ho, Thanos Karatzias, Mark Shevlin, Kwan Ho Wong, Philip Hyland

**Affiliations:** 1 Department of Psychiatry, Chaohu Hospital of Anhui Medical University, Hefei, China; 2 School of Mental Health and Psychological Sciences, Anhui Medical University, Hefei, China; 3 Anhui Psychiatric Center, Anhui Medical University, Hefei, China; 4 School of Nursing, The Hong Kong Polytechnic University, Kowloon, Hong Kong; 5 School of Health & Social Care, Edinburgh Napier University, Edinburgh, UK; 6 NHS Lothian, Rivers Centre for Traumatic Stress, Edinburgh, UK; 7 School of Psychology, Ulster University, Derry, Northern Ireland; 8 Department of Psychology, Maynooth University, Maynooth, Ireland

**Keywords:** ICD-11, CPTSD, Self-harm, Suicide, Adolescents, Youths

## Abstract

Posttraumatic stress disorder (PTSD) is linked with self-harm and suicide, but few studies have examined these severe outcomes in relation to complex trauma. This study examined the associations between self-harm and suicide-related phenomena with ICD-11 complex PTSD (CPTSD) among treatment-seeking youths. A convenience sample of 109 adolescents with major depression (69.7% female; mean age = 15.24) were recruited from an outpatient psychiatric clinic. Participants completed measures for ICD-11 CPTSD, adverse childhood experiences (ACEs), self-harm behaviors, and past-year history of four suicide-related phenomena. Relationships between each self-harm and suicide-related variable with CPTSD were assessed at the symptom and diagnostic levels. Participants reported an average of three ACEs; 33.9% met diagnostic requirements for ICD-11 CPTSD. Past-year suicidal thought and attempt, but not self-harm, significantly associated with CPTSD status. At the symptom level, self-harm associated with CPTSD total symptom and all symptom clusters scores, with strongest associations found with symptoms of negative self-concept. CPTSD total symptom scores also associated strongly with past-year history of suicidal thought, plan, and attempt; the three core PTSD symptom clusters scores consistently and strongly linked with these suicide-related phenomena. For symptoms of complex trauma, relationship disturbances associated with having a suicide attempt, and negative self-concept associated with both having a plan and an attempt. Assessing and targeting ICD-11 CPTSD symptoms have potential to reduce self-harm and suicidality in young people experiencing mental distress, particularly for those with a trauma history and regardless of whether they meet criteria for a diagnosable trauma response.

Youth self-harm and suicide is a serious public health concern linked with substantial adverse socioeconomic effects, healthcare burden, and societal loss (World Health Organization, 2021). Self-harm, defined as purposeful and intentional self-destructive behaviors without suicidal intention (Nock et al., 2010), is an increasing phenomenon globally among youths, with an estimated prevalence of 17.2% among adolescents (Swannell et al., 2014). Although self-harm can occur independent of suicide (Hawton & James, 2005), studies suggest self-harm increases the risk of subsequent suicidal behaviors (Beckman et al., 2016; Morgan et al., 2017). Recent estimates showed suicide is the 4th leading cause of death in youths aged 15–29 years worldwide (World Health Organization, 2021). The rates of first onset of suicidal thoughts and attempts are also highest among adolescents and young adults (Bernal et al., 2007). A systematic review and meta-analysis of 365 studies of youths between ages 6 to 21 years from ten global regions found between 14 and 23% had active suicidal ideations, 4-23% had made plans for suicide, and 5-15% had a suicide attempt (Van Meter et al., 2022).

Posttraumatic stress disorder (PTSD) is linked with functional impairment, mental health disturbances, and elevated risks of self-harm and suicide (Tull et al., 2016). A meta-analysis of 72 studies estimated around 15.9% of trauma-exposed adolescents will develop PTSD (Alisic et al., 2014), and another study found approximately 5% of adolescents meet PTSD criteria in their lifetime (Merikangas et al., 2010). Many studies have demonstrated a positive correlation between PTSD and self-harm (Ford & Gómez, 2015; Sami & Hallaq, 2018; Viana et al., 2017; Zhao et al., 2023). PTSD is associated with greater difficulties in emotion regulation and higher levels of impulsivity, thereby increasing the risk of engaging in self-harm behaviors (Tull et al., 2016). For example, emotional regulation theory (Nock, 2009) and the cognitive-emotional model on non-suicidal self-injury (Hasking et al., 2017) both conceptualize self-harm behaviors as a maladaptive emotion regulation strategy to cope with the experiences of PTSD symptoms (Zhao et al., 2023). Indeed, many studies have found a robust association between self-harm behaviors and PTSD (Ford & Gómez, 2015; Sami & Hallaq, 2018; Zhao et al., 2023), with some evidence showing depressive symptoms mediate the relationship between trauma exposure and self-harm in treatment-seeking youths (Zhou et al., 2023).

Numerous studies demonstrated PTSD is also a critical risk factor for suicidality among trauma-exposed youths (Eskander et al., 2020; Nock et al., 2013; Waldrop et al., 2007), with results from one systematic review and meta-analysis of 28 studies showing significant associations between PTSD and different suicide-related phenomena, including suicidal thoughts, plans, and attempts, among adolescents in both treatment-seeking and community samples (Panagioti et al., 2015). Further, PTSD is more strongly associated with suicidality compared with other psychiatric conditions. For example, Wunderlich et al. (1998) found the risk of suicide attempts is highest among those diagnosed with PTSD (OR = 7.8) compared with substance disorder (OR = 2.2) and depressive disorder (OR = 2.1). Another study of 159,500 adolescent inpatients in the U.S. (Eskander et al., 2020) found the diagnosis of PTSD increased suicidality risk by 23% when compared with non-PTSD psychiatric adolescent inpatients. Importantly, the risk of suicide is potentiated when PTSD is comorbid with other psychiatric conditions, especially depression (Krysinska & Lester, 2010; Oquendo et al., 2005; Panagioti et al., 2012b). This may be explained by the overlapping emotional and cognitive vulnerabilities commonly shared across both conditions, such as hopelessness (Panagioti et al., 2012a), negative appraisals of defeat and entrapment (Panagioti et al., 2013), and emotion dysregulation and interpersonal factors (Díaz-Oliván et al., 2021).

Repeated exposure to adverse childhood experiences (ACEs) and interpersonal trauma is associated with a higher risk of comorbid psychiatric conditions and more severe psychopathology (Cloitre et al., 2013; Ho et al., 2021; Hyland et al., 2017). The 11th version of the International Classification of Diseases (ICD-11) (World Health Organization, 2018) introduced a new diagnosis of complex posttraumatic stress disorder (CPTSD) to capture more complex trauma reactions (Hyland et al., 2018a). According to the ICD-11 diagnostic formulation, CPTSD includes the core PTSD symptom clusters (i.e. re-experiencing, avoidance, and hyperarousal) and three additional symptom clusters (i.e. emotional dysregulation, interpersonal difficulties, and negative self-concept) that were collective labeled ‘disturbances in self-organization’ (DSO). Existing studies support PTSD and CPTSD as separate diagnoses (Cloitre et al., 2018b; Ho et al., 2020; Hyland et al., 2017; Karatzias et al., 2017; Redican et al., 2021), and that CPTSD is distinguishable from PTSD in different samples of traumatized adolescents (Haselgruber et al., 2020; Hebert & Amedee, 2020; Kazlauskas et al., 2020). In the context of adversities that occurred during childhood, individuals with ACEs were more likely to meet the diagnostic requirements of ICD-11 stress-related disorders (Ho et al., 2019b), with evidence showing those with higher cumulative exposure to ACEs had higher odds of screening positive for ICD-11 CPTSD than PTSD (Karatzias et al., 2017). Results from several studies (Ho et al., 2019a, c; Hyland et al., 2017) also showed that PTSD and DSO symptoms differentially link with different exogenous mental health states; depression was significantly linked with DSO but not PTSD, while anxiety was associated with both but more strongly with PTSD. Compared with ICD-11 PTSD, ICD-11 CPTSD is also more likely to co-occur with other psychiatric conditions, and is associated with more impairments in functioning, quality of life, and mental disturbances, such as depressive, anxiety, dissociative symptoms (Brewin et al., 2017; Hyland et al., 2018b; Karatzias & Cloitre, 2019; Karatzias et al., 2017, 2019c; Murphy et al., 2021). These findings suggest ICD-11 CPTSD is a more severe condition, and that different clinical interventions and treatment targets are needed for young clients experiencing PTSD versus CPTSD (Karatzias & Cloitre, 2019; Karatzias et al., 2019b).

To date, the associations between ICD-11 CPTSD and self-harm and suicide remain unclear, and the empirical evidence is still absent in adolescents. Results from previous studies have shown that CPTSD was independently associated with younger age and exposure to interpersonal and childhood trauma (Karatzias et al., 2019), and that it is strongly associated with suicidality (Gelezelyte et al., 2022; Hyland et al., 2018a; Karatzias et al., 2019c; Møller et al., 2021; Spikol et al., 2022), but these studies were conducted in adult samples and in Western countries. We are unaware of any study that has examined the relationships between ICD-11 CPTSD and self-harm and suicide-related phenomena among adolescents or in Asia. Further, no known study has assessed how ICD-11 CPTSD is linked to self-harm and suicidality at the symptom level. A more precise determination of how CPTSD is related to these severe outcomes among youths will be essential for informing its identification, treatment, and management in this vulnerable group.

The present study was the first to examine the relationships between ICD-11 CPTSD and multiple self-harm and suicide related phenomena among youths. In a clinically high-risk sample of Chinese adolescents, the first objective of this study was to determine if meeting diagnostic criteria for ICD-11 CPTSD was positively associated with deliberate self-harm behavior and past-year history of having suicidal thoughts, a suicide plan, attempted suicide, and attempted suicide that resulted in medical intervention. The second objective was to explore these relationships at the symptom level rather than the diagnostic level, and to determine if specific symptom clusters of ICD-11 CPTSD were especially strongly correlated with these different self-harm and suicide-related phenomena.

## Methods

### Participants and Procedures

This cross-sectional survey study recruited participants by convenience sampling at one psychiatric outpatient clinic attached to a major University hospital located in an eastern province of Mainland China. Screening and referrals were made by participants’ corresponding physicians. Participants included adolescents between ages 12–17 years, receiving psychiatric care at the clinic and in a stable condition, and endorsed by their physician to participate. Adolescents diagnosed with multiple psychiatric and/or comorbid physical health conditions were excluded. The final sample consisted of 109 Chinese adolescents (69.7% female; mean age = 15.24, SD = 1.45) diagnosed with major depressive disorder and completed all study measures.

The study was approved by the ethics committee of the first author’s affiliated institution. Written parental/guardian consent, adolescents’ assent, and endorsement from their corresponding physician were obtained before survey completion. Participants completed the paper-and-pencil surveys independently in a private area at the clinic. Questionnaires were collected by a research team member who was not a part of the adolescents’ clinical care team.

### Measures

*ICD-11 CPTSD* was assessed using the Chinese version of the International Trauma Question – Child and Adolescent version (ITQ-CA) (Cloitre et al., 2018a; Ho et al., 2022), a 22-item self-report measure of ICD-11 PTSD and CPTSD for people aged 7–17 years. The measure includes 6 items that reflect three PTSD symptom clusters: ‘Re-experiencing’, ‘Avoidance’, and ‘Sense of Threat’, and 6 items that reflect three DSO symptom clusters: ‘Affective Dysregulation’, ‘Negative Self-Concept’, and ‘Disturbed Relationships’. Participants first reported their exposure to 13 types of adverse childhood experiences (ACEs) using the Chinese ACE - International Questionnaire (Ho et al., 2019a, c). Then, participants responded to items on the ITQ-CA by identifying an event that was currently bothering them the most and indicate how much they were bothered by the symptoms in the past month using a 5-point Likert scale ranging from ‘Not at all’ (0) to ‘Extremely’ (4). Functional impairment was separately assessed for PTSD and DSO using five additional items: interference with friendship, family relationship, school work, other important life aspects, and general happiness.

Probable caseness of ICD-11 PTSD is defined as endorsement of ‘Moderately’ (2) or above for at least one symptom in each PTSD symptom cluster and responding ‘yes’ to at least one functional impairment item related to those symptoms. Probable caseness of ICD-11 CPTSD is defined as meeting criteria for PTSD, and endorsement of ‘Moderately’ (2) or above for at least one symptom in each DSO symptom cluster and responding ‘yes’ to at least one functional impairment item related to the DSO symptoms. An individual can meet requirements for PTSD or CPTSD, but not both. The internal consistency of the 12 core items in the present sample was .87.

*Self-harm* was measured using the 17-item Deliberate Self-Harm Behavior Scale (Shek & Yu, 2012), which measures the occurrence (i.e. yes/ no) of 17 different self-harming behaviors in the past year, such as wrist cutting; burning, scratching, or biting oneself; and preventing wounds from healing. To differentiate from suicide-related behaviors, participants were reminded to respond ‘yes’ only if the self-harm behavior was carried out without suicidal intention. Endorsements were summed to create a composite score ranging from 0 to 17 with higher scores indicating more self-harming behaviors. The internal consistency for the present sample was .85.

*Suicide-related phenomena* were measured with four separate items. Respondents were asked if they had (1) any suicidal thoughts, (2) made a plan for suicide, (3) had a suicide attempt, and (4) had a suicide attempt that resulted in medical intervention. Responses were based on whether any of the above were present (yes/ no) within the past year.

### Data Analysis

Descriptive statistics summarized ACE exposure; occurrence of different deliberate self-harming behaviours and suicide-related phenomena; and ICD-11 PTSD and CPTSD. Associations between ICD-11 CPTSD symptoms and sex and age were assessed via an independent samples t-test and a Pearson product moment correlation test, respectively. Associations between meeting diagnostic criteria for ICD-11 CPTSD and sex and age were assessed via a Pearson chi-square test of association, and an independent samples t-test. Cohen’s d values and odds ratios were calculated to estimate effect sizes. An independent samples t-test was used to compare levels of self-harm between those that did and did not meet diagnostic requirements for ICD-11 CPTSD. Pearson chi-square tests were used to assess the association between meeting diagnostic criteria for ICD-11 CPTSD and having past-year history of suicidal thoughts, a suicide plan, a suicide attempt, and a suicide attempt requiring medical intervention. Pearson product-moment correlation tests were used to assess the association between self-harm with ICD-11 CPTSD total and symptom cluster scores. Last, independent samples t-tests were used to compare mean levels of total and symptom cluster scores of ICD-11 CPTSD across the four suicide-related variables.

## Results

### Descriptive Statistics

Among 109 Chinese adolescents diagnosed with major depression, the average number of ACEs was 3.00 (SD = 2.45); 64.2% (*n* = 70) had two or more ACEs. The most common ACE was physical abuse (56.9%), followed by emotional abuse (47.7%), witnessing domestic violence (38.5%), parental death or separation (35.8%), emotional neglect (29.4%), and sexual abuse (24.8%).

The mean number of deliberate self-harming behaviours was 4.39 (Mdn = 4.00, SD = 3.45, range 0–14); 75.2% (*n* = 82) had a past-year history of suicidal thoughts, 55.0% (*n* = 60) indicated a suicide plan in the past year, 57.8% (*n* = 63) made a suicide attempt in the past year, and 10.1% (*n* = 11) required medical intervention as a result of their suicide attempt.

At the diagnostic level, a total of 42.2% (*n* = 46) met diagnostic requirements for either ICD-11 PTSD or CPTSD; 33.9% met criteria for CPTSD (*n* = 37) and 8.3% (*n* = 9) met criteria for PTSD. A higher proportion of females met criteria for ICD-11 CPTSD than males (38.2% vs. 24.2%), but this effect was not statistically significant (OR = 1.93, χ2 (1) = 1.99, *p* = .159). There was no significant difference in the mean age of those that did (M = 15.03, SD = 1.62) and did not (M = 15.35, SD = 1.34) meet diagnostic criteria for ICD-11 CPTSD (t (107) = 1.10, *p* = .276, d = 0.18).

At the symptom level, the mean CPTSD total score was 27.84 (Mdn = 29.00, SD = 9.56, range = 1–46), with females (M = 29.30, SD = 8.51) scoring significantly higher than males (M = 24.48, SD = 11.03); t (107) = 2.48, *p* = .015, d = 0.52). There was no significant association between CPTSD total scores and age (*r* = − .15, *p* = .124).

### Associations between ICD-11 CPTSD and Self-harm and Suicide

There was no significant difference in the mean number of deliberate self-harming acts between those that did (M = 5.27, SD = 3.22) and did not (M = 3.94, SD = 3.50) meet diagnostic criteria for ICD-11 CPTSD (t (107) = 1.93, *p* = .057, d = 0.39). However, a significant, positive, and moderate association between the number of deliberate self-harming acts and ICD-11 CPTSD total scores (*r* = .47, *p* < .001) was observed. At the symptom level, self-harm behaviours also significantly correlated with all CPTSD symptom clusters: re-experiencing (*r* = .36, *p* < .001), avoidance (*r* = .34, *p* < .001), sense of current threat (*r* = .34, *p* < .001), affective dysregulation (*r* = .27, *p* = .007), negative self-concept (*r* = .41, *p* < .001), and disturbed relationships (*r* = .32, *p* < .001).

Table 1 presents the proportions of adolescents meeting diagnostic criteria for ICD-11 CPTSD for each suicide-related variable. Those with a past-year history of suicidal thoughts and attempt were significantly more likely to meet diagnostic requirements for ICD-11 CPTSD. Specifically, those indicating suicidal thoughts in the past year had 5.67 times higher odds of meeting requirements for ICD-11 CPTSD; those who attempted suicide in the last year had 4.05 times higher odds of meeting requirements for ICD-11 CPTSD.

**Table 1:** Differences in the proportion of adolescents reporting suicide-related phenomena based on ICD-11 CPTSD status

		CPTSD	χ^2^	OR (95% CI)
Suicidal thoughts	No Yes	8.1% 41.5%	8.35***	5.67 (1.58, 20.34)
Suicide plan	No Yes	26.5% 40.0%	2.18	1.85 (0.82, 4.18)
Suicide attempt	No Yes	17.4% 46.0%	9.73***	4.05 (1.63, 10.06)
Suicide attempt - medial intervention	No Yes	33.7% 36.4%	0.03	1.13 (0.31, 4.12)

Note: χ^2^ = Pearson chi-square test; all degrees of freedom = 1; OR (95% CI) = Odds ratio with 95% confidence intervals

Table 2 presents the mean levels of ICD-11 CPTSD symptoms across each of the suicide-related variables. Adolescents indicating a past-year history of suicidal thoughts, a suicide plan, and a suicide attempt had significantly higher ICD-11 CPTSD symptom scores, and all differences were of a large magnitude with Cohen’s d values > 1.08. The difference in mean levels of ICD-11 CPTSD symptoms between those requiring and not requiring medical attention following a suicide attempt was large (d = 1.18) but not statistically significant.

**Table 2:** Mean differences in ICD-11 CPTSD total symptom scores across suicide-related phenomena

		*N*	Mean	SD	t-value	Cohen’s d
Suicidal thoughts	No Yes	27 82	18.74 30.84	9.24 7.59	6.80***	1.51
Suicide plan	No Yes	49 60	24.39 30.67	10.06 8.18	3.60***	1.08
Suicide attempt	No Yes	46 63	22.98 31.40	9.97 7.52	5.03***	1.38
Suicide attempt with medical intervention	No Yes	98 11	27.32 32.55	9.44 9.74	1.74	1.18

Note. SD = standard deviation; statistical significance = * *p* < .05, ** *p* < .01, ***p < .001

Those with and without suicidal thoughts, a plan, an attempt, and an attempt requiring medical intervention were also compared on their mean levels of each ICD-11 CPTSD symptom cluster (see Table 3). Those with suicidal thoughts had significantly higher levels of each symptom cluster and all effects were large, ranging from d = 1.09 (affective dysregulation) to d = 1.71 (negative self-concept). Those reporting a suicide plan had significantly higher levels of re-experiencing, avoidance, sense of current threat, and negative self-concept. The largest effects were for avoidance (d = 1.11) and sense of current threat (d = 1.13). Those who had a suicide attempt had significantly higher levels of all symptom clusters except affective dysregulation, and the largest effect was for re-experiencing (d = 1.22). Those with a suicide attempt that resulted in medical intervention significantly differed only on their symptoms of re-experiencing (d = 1.60).

**Table 3:** Mean differences across all ICD-11 CPTSD symptom clusters across suicide-related phenomena

	Suicide thoughts	Suicide plan	Suicide attempt	Suicide attempt with medical intervention
	t-value (d)	t-value (d)	t-value (d)	t-value (d)
Re-experiencing	3.64*** (1.25)	2.49* (0.48)	4.25*** (1.22)	3.05** (1.60)
Avoidance	5.04*** (1.58)	3.76*** (1.11)	3.91*** (1.15)	1.39 (1.07)
Sense of current threat	5.43*** (1.67)	3.89*** (1.13)	5.05*** (0.98)	1.47 (1.09)
Affective dysregulation	2.92** (1.09)	1.18 (0.23)	1.85 (0.74)	0.10 (0.65)
Negative self-concept	5.66*** (1.71)	2.97** (0.57)	3.59*** (1.09)	1.49 (1.10)
Disturbed relationships	4.52*** (1.46)	1.25 (0.24)	2.50* (0.87)	0.18 (0.68)

Note. d = Cohen’s d; Statistical significance: * *p* < .05, ** *p* < .01, ***p < .001

## Discussion

The current study was the first to examine the relationships between ICD-11 CPTSD and self-harm and suicide in a high-risk sample of mental health treatment-seeking adolescents. Participants reported an average of three ACEs, which suggests this sample of youths had multiple serious and potentially traumatic adversities in early life. These findings corroborate with emerging research on ACEs in Chinese youths showing that ACE exposure is common and associated with depressive symptoms in a dose-response fashion (Jiang et al., 2022; Zhang et al., 2020). In line with previous research with young adults from the general population (Kazlauskas et al., 2022; Redican et al., 2022) and clinical samples (Møller et al., 2020), our findings indicate a higher proportion of participants met criteria for CPTSD (33.9%) than PTSD (8.3%). However, compared to a previous study of community dwelling young adults in East Asia (Ho et al., 2020), our clinical sample had significantly higher rates of meeting diagnostic requirements for ICD-11 CPTSD (33.9% versus 3.6%). Given approximately half of people with PTSD were estimated to also have a comorbid diagnosis of major depressive disorder (Flory & Yehuda, 2015), it was not surprising that the present sample with an existing diagnosis of depression also had high rates of a diagnosable trauma reaction. The high rate of CPTSD in the present sample may also be explained by findings from prior research (D’Andrea et al., 2012; Hyland et al., 2017; Park et al., 2013) showing that symptoms of DSO are significantly associated with depression and exposure to trauma in childhood, and are more commonly endorsed in East Asian cultures regardless of PTSD presentation. Specific to the Chinese cultural context, results of previous research also showed emotional difficulties was a salient dimension of developmental trauma in Chinese children (Ma & Li, 2014), and that affective dysregulation (a DSO symptom) correlated highly with all PTSD symptom clusters (Ho et al., 2019a, c).

Our first objective was to determine if meeting diagnostic criteria for ICD-11 CPTSD was positively associated with deliberate self-harm behavior and past-year history of suicidal thoughts, having a suicide plan, having attempted suicide, and having attempted suicide that resulted in medical intervention. At the diagnostic level, we did not find significant associations between CPTSD and self-harm, which contradicts previous findings (Hyland et al., 2018a). Given the strong relationship between depression and self-harm (Klonsky, 2007; Marshall et al., 2013), it is possible that our clinical sample did not provide sufficient variability in levels of self-harming behaviors to distinguish respondents with ICD-11 CPTSD at the diagnostic level. However, in line with previous research in general and trauma-exposed adults (Karatzias & Cloitre, 2019; Karatzias et al., 2019c), meeting diagnostic requirements for ICD-11 CPTSD was significantly associated with higher odds of having past-year suicidal thought and attempt. These findings extends previous research on PTSD, depression, and suicidality among adolescents (Panagioti et al., 2015), and further suggest complex presentations of trauma reactions and suicide risks are high in young people with depression. Therefore, suicide risk and co-occurrence of CPTSD must be routinely assessed in clinical settings serving youths seeking treatment for their depressive symptoms.

Our second objective was to examine the associations between self-harm and suicide with ICD-11 CPTSD at the symptom level. Although CPTSD did not associate with self-harm behaviors at the diagnostic level, associations emerged at the symptom level such that self-harm behaviors were moderately and positively associated with CPTSD total scores and all symptom cluster scores, with the strongest association found with symptoms of negative self-concept. These findings suggest CPTSD relates to self-harm more so at the symptom level rather than the diagnostic level. Given the clear link between depression and self-harm, and the context of our present sample, more research is needed to examine these relationships when accounting for other mental disorders and comorbid conditions. Nonetheless, our results underscore the importance of routinely assessing risks and presence of self-harm and suicide-related phenomena even in the absence of CPTSD diagnosis in trauma-exposed youths.

Limited studies have examined how ICD-11 CPTSD is associated with suicide at the symptom level. We found that total CPTSD symptom scores were associated with having suicidal thoughts, a suicide plan, and suicide attempts with large effects, and all symptom clusters strongly associated with having suicidal thoughts in the past year. Consistent with prior work (Batterham et al., 2018; Bayliss et al., 2022), our findings showed PTSD symptoms contributed consistently and strongly to the transition from suicidal ideation to action (i.e. plan and attempt). In fact, re-experiencing was the only symptom associated with having a suicide attempt requiring medical intervention. For DSO, we did not observe a relationship between affect dysregulation scores and past-year history of suicide plan or attempt, but this should be interpreted with caution given it is likely that our sample of adolescents with major depression all experienced significant challenges with emotion regulation. Conversely, disturbed relationship scores were associated with having a suicide attempt, and negative self-concept scores were associated with both having a plan and having made an attempt. Taken together, our results showed that, at least for those with major depression, relationship disturbances and poor sense of self are additional salient factors related to higher-risk suicide-related phenomena in treatment-seeking youths. Indeed, low belongingness, poor social connection, and hopelessness are significant risk factors that can turn suicidal thoughts into actions (Klonsky & May, 2015; Van Orden et al., 2010). Recent theoretical explanations for ICD-11 CPTSD (Hyland et al., 2023) also posits traumatic life events causes disruptions in identities, and that negative self-concept may be especially strongly related to negative identities marked by worthlessness and inferiority, which are strongly linked with suicidal behavior (Butter et al., 2019; Panagioti et al., 2012). In fact, one study found that negative self-concept was the only ICD-11 CPTSD symptom associated with suicidality (McGinty et al., 2023). Although this study was conducted with a representative adult sample, some evidence highlights negative self-concept as a salient characteristic of CPTSD (Karatzias & Cloitre, 2019), especially for those with poly-traumatization that occur in childhood (Karatzias et al., 2020). Overall, our findings support negative self-concept as a key risk for self-harm and suicide among treatment-seeking youths. More research is needed to understand whether and how negative self-concept mechanistically impact the development of self-harm and suicide so as to prevent these catastrophic outcomes in this highly vulnerable group.

Several study limitations should be considered. First, the small, homogenous sample recruited from one outpatient psychiatric clinic in Mainland China limits the generalizability of findings. Second, the cross-sectional nature of the study precludes drawing causal inferences between CPTSD with self-harm and suicide. Third, there may be other confounding factors, such as social support or additional trauma history besides ACEs, that were not included. Last, the use of self-report measures in the clinical setting may impart response or social desirability biases. In particular, sensitive information on trauma histories (e.g. abuse) and experiences with self-harm or suicide collected in the current setting may have led to underreporting. However, some evidence supports the stability of retrospective self-reported ACEs in Chinese young people using the ACE-IQ (Ho et al., 2019a, c).

## Conclusion

The current study found over 1-in-3 treatment-seeking adolescents diagnosed with major depressive disorder met diagnostic requirements for ICD-11 CPTSD. ICD-11 CPTSD was positively associated with self-harm and suicide at the symptom level, and CPTSD also associated with suicidal thought and attempt at the diagnostic level. Therefore, early identification of CPTSD and assessing CPTSD symptoms as a means to identify risks for self-harm and suicide have potential to prevent these severe outcomes in this vulnerable population. Specifically, our results showed that, in addition to symptoms of PTSD, negative self-concept was a key CPTSD symptom cluster correlated with self-harm and suicide-related phenomena. This highlights the importance for clinicians to routinely screen for these symptoms in treatment-seeking adolescents experiencing significant mental distress, particularly those with a trauma history and regardless of whether they meet criteria for a diagnosable trauma response. Our results also suggest that targeting CPTSD symptoms might reduce self-harm and suicidality in young people. There is limited evidence at present on effective treatments for CPTSD in adults and younger people, and there is clearly a need to develop and test evidence-based treatments (Karatzias et al., 2019a). Continued investigations are needed to clarify the relationships between ICD-11 CPTSD and self-harm and suicide in different clinical and general youth populations across cultures.

## Abbreviations

ICD-11: 11th version of the International Classification of Diseases
PTSD: Posttraumatic stress disorder
CPTSD: Complex posttraumatic stress disorder
ACE: Adverse childhood experience
